# Analytical characterization of trace elements (zinc, copper, cadmium, lead and selenium) in saliva of pigs under common pathological conditions in the field: a pilot study

**DOI:** 10.1186/s12917-020-2245-6

**Published:** 2020-01-30

**Authors:** Jorge Sánchez, Miguel Montilla, Cándido Gutiérrez-Panizo, Juan Sotillo, Pablo Fuentes, Ana Montes, Ana María Gutiérrez

**Affiliations:** 1 BioVetMed Research Group, Department of Animal Medicine and Surgery, Veterinary School, University of Murcia, Espinardo, 30100 Murcia, Spain; 2 Cefu S.A., 30840 Alhama de Murcia, Murcia Spain

**Keywords:** Zn, Cu, Pd, Cd, Se, Salivary levels, Disease detection

## Abstract

**Background:**

This study is focused on the measurement of trace elements (zinc, copper, cadmium, lead and selenium) in the saliva of pigs in order to study their levels on different porcine pathological conditions in the field. The experiment involved 15 pigs without clinical signs of disease and 42 diseased pigs (suffering from lameness, rectal prolapse, fatigue or growth rate retardation). Individual saliva samples were collected, allowing the pigs to chew a sponge each for trace element quantifications through atomic absorption spectrometry (AAS). Since this is the first report on the measurements of trace elements in porcine saliva, a routine analytical validation study was performed for the quantification of all the studied elements. Moreover, the acute phase proteins C-reactive protein (CRP) and haptoblobin (Hp), the total antioxidant capacity (TAC) and adenosine deaminase (ADA) were quantified in the saliva samples for the animal’s health status assessment.

**Results:**

Modifications in the levels of acute phase proteins or ADA were only recorded in animals with lameness and rectal prolapse and those with fatigue respectively. Moreover, TAC level changes were observed in pigs with growth-rate retardation. However, alterations in the levels of two or more trace elements were reported for all the different groups of diseased pigs with evident variations within pathologies.

**Conclusions:**

The salivary quantification of trace elements could be considered as a complementary tool to acute phase proteins, TAC and ADA determinations for disease detection and differentiation in the pig and should be explored in greater depth.

## Background

Over the past few years, the use of saliva samples in pigs for diagnosis purposes has been expanded due to its several advantages. The easy, quick, non-invasive and economic sampling allowing unskilled people to collect many samples even in remote places and with minimal equipment [1] makes saliva a good option as an alternative diagnostic fluid.

In addition, saliva analysis can reflect systemic conditions in pigs as previously stated [2]. Studies regarding acute phase proteins (APPs) in pigs were based on the C-reactive protein (CRP), haptoglobin (Hp) and serum amyloid A (SAA), indicating that salivary APPs concentration can be used as an early indicator of health status [3, 4]. Moreover, recent studies performed over the enzyme adenosine deaminase (ADA) in saliva samples showed a powerful and cheap tool for health status assessment in pigs [5]. Other salivary biomarkers such as α-amylase [6], cortisol, chromogranin A, immunoglobulin A or testosterone [7] could be used as a practical and non-invasive tool for reflecting the activity of different physiological systems involved in the stress response and the immune system in pigs [8]. Furthermore, a pen-based collection of oral fluids has been used to monitor the circulation of several pathogens, such as the porcine reproductive and respiratory virus (PRRS) [9], porcine circovirus Type 2 [10], influenza A virus [11] and *Erysipelothrix rhusiopathiae* [12].

As such, the importance of measuring trace elements is based on their own roles in the organism (immune system, enzymatic co-factors or cellular structure among others), related to the health status and the interactions between the elements themselves and the changes produced in a diseased organism. Copper (Cu) and zinc (Zn) are essential trace elements for metabolic functions in mammals’ cells, both being co-factors for enzymes. Cu participates in the activation of oxidative enzymes are required for normal cellular metabolism, while Zn is a cofactor in more than 300 metalloenzymes, which inhibits some bacterial populations in the intestinal tract [13]. Another crucial role of Zn is that it acts as an integral part of the host immune response by limiting Zn pathogens’ availability [14]. Additionally, salivary Cu and Zn quantifications, as reported, have a potential power for the diagnosis of human malignant lesions in the oral cavity [15].

Studies on the experimental infection of pigs with *Actinobacillus pleuropneumoniae* have shown an increase in the levels of Cu and decrease in the concentrations of Zn in plasma after 4 days of infection [16]. Moreover, other studies have suggested that pathogens can have a competitive advantage over the commensal microbiota under Zn limiting conditions, thereby being promoted under an inflamed state [17]. Subsequently, the effects of zinc over humoral and cellular immune responses are well recognised in pigs. Zn enhances the immune response to infection and leads to a decreased number and severity of lesions [18].

A role in oxidative stress development has also been reported for some trace elements. Accordingly, Zn acts as a cofactor for Cu-Zn superoxide dismutase enzyme that is a part of the primary antioxidant system of all vertebrates [19]. Furthermore, selenium (Se) participates in several enzymatic reactions in pigs, such as in antioxidant defence mechanism and the inhibition of viral replication [20], while lead (Pb) induces oxidative stress in tissues and cellular components, causing damage to membranes, DNA and proteins [21]. The mechanism underlying lead-induced oxidative damage to membranes is associated with changes in its fatty acid composition [22]. In addition, cadmium (Cd) stimulates the formation of reactive oxygen species, thus causing oxidative damage to erythrocytes and tissues resulting in a loss of membrane functions [23].

The present study aims to perform, for the first time, the quantification of trace elements contained in swine saliva by atomic absorption spectrometry (AAS), and show its potential contribution to the detection and differentiation of health status alterations in pigs in field conditions.

## Results

### Analytical validation

The all intra-assay coefficient of variation (CV) for the pools with high and low levels of trace elements studied were lower than 9% (Table 1). Moreover, the inter-assay CV obtained for all the trace elements were lower than 9.6% (Table 2).

**Table 1:** Assessment of the intra-assay precision of the FAAS assay for the measurement of Zn, Cu, Cd, Pb and Se in saliva samples of pigs

Measurements		Mean (SD)	CV (%)	Overall CV (%)
Zn (μg/mL)	High content	3.10 (0.21)	6.68	5.02
	Low content	0.35 (0.01)	3.36	
Cu (μg/mL)	High content	0.30 (0.02)	8.29	8.14
	Low content	0.15 (0.012)	7.99	
Cd (ng/mL)	High content	1.28 (0.041)	3.21	5.96
	Low content	0.33 (0.028)	8.71	
Pb (ng/mL)	High content	39.55 (1.123)	2.84	5.35
	Low content	2.45 (0.193)	7.85	
Se (ng/mL)	High content	7.90 (0.634)	8.02	6.75
	Low content	3.15 (0.173)	5.49

**Table 2:** Assessment of the inter-assay precision of the FAAS assay for the measurement of Cu, Zn, Cd, Pb and Se in saliva samples of pigs

	Zn (μg/mL)	Cu (μg/mL)	Cd (ng/mL)	Pb (ng/mL)	Se (ng/mL)
Mean	1.07	0.97	0.93	10.30	9.12
SD	0.067	0.013	0.089	0.743	0.431
CV (%)	6.26	1.41	9.57	7.21	4.72

**Fig. 1:**
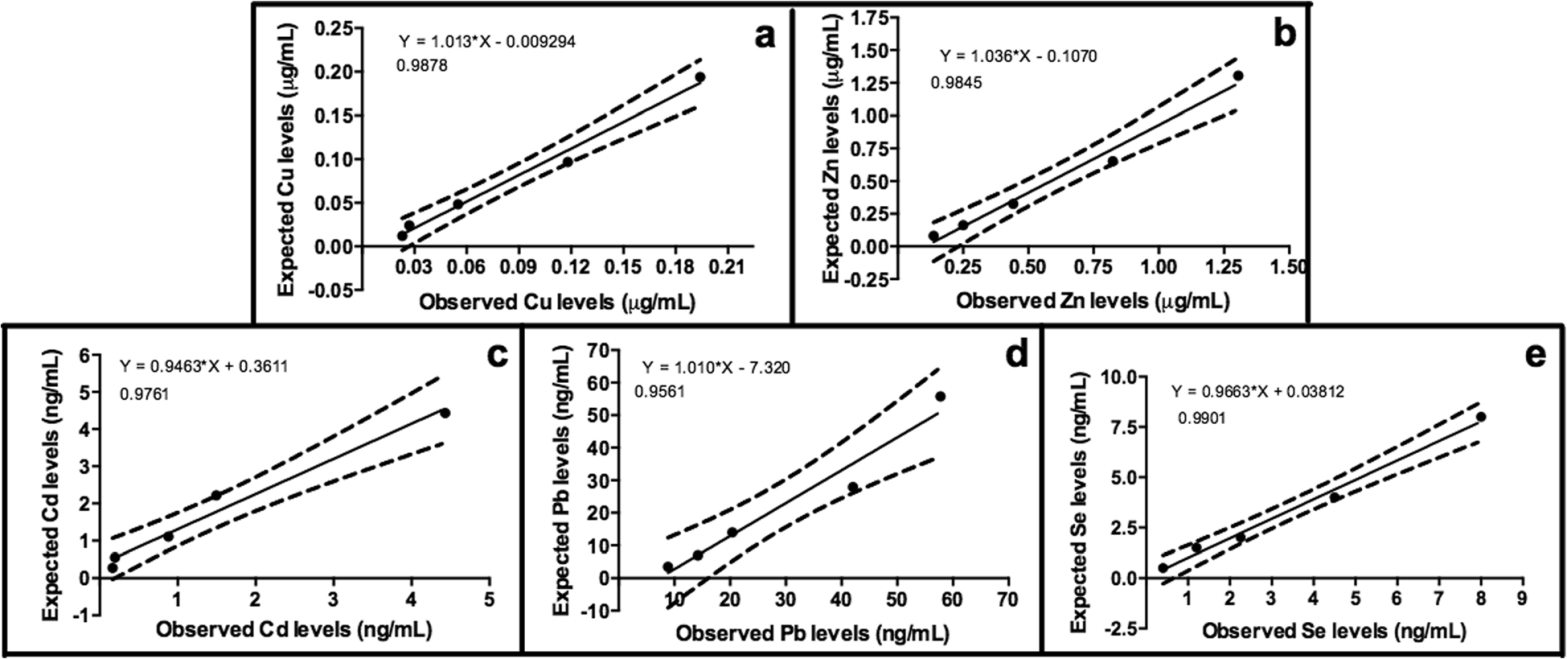
Linear regression lines indicating the accuracy of the different assay used for measuring Cu (**a**), Zn (**b**), Cd (**c**), Pd (**d**) and Se (**e**) in serial dilutions of a saliva sample from swine. The slope of the regression lines and the intercepts are indicated in the equation of the curve (*R* square = correlation coefficients)

The dilution of 2 saliva samples with high concentrations of the trace elements resulted in linear regression equations where x represents the expected trace element level at the dilution and y represents the measured level of the trace element. The correlation coefficients were 0.98 for Cu and Zn, 0.97 for Cd, 0.95 for Pb and 0.99 for the Se measurements (Fig. 1).

The limit of detection in the Cu and Zn measurements was 0.005 μg/mL and 0.012 μg/mL respectively, while those of 0.015 ng/mL, 0.539 ng/mL and 0.582 ng/mL were obtained for Cd, Pb and Se quantifications respectively. Additional file 1 provides the raw data of the analytical validation study of trace elements in porcine saliva samples.

### The measurements of trace elements under clinical conditions

**Fig. 2:**
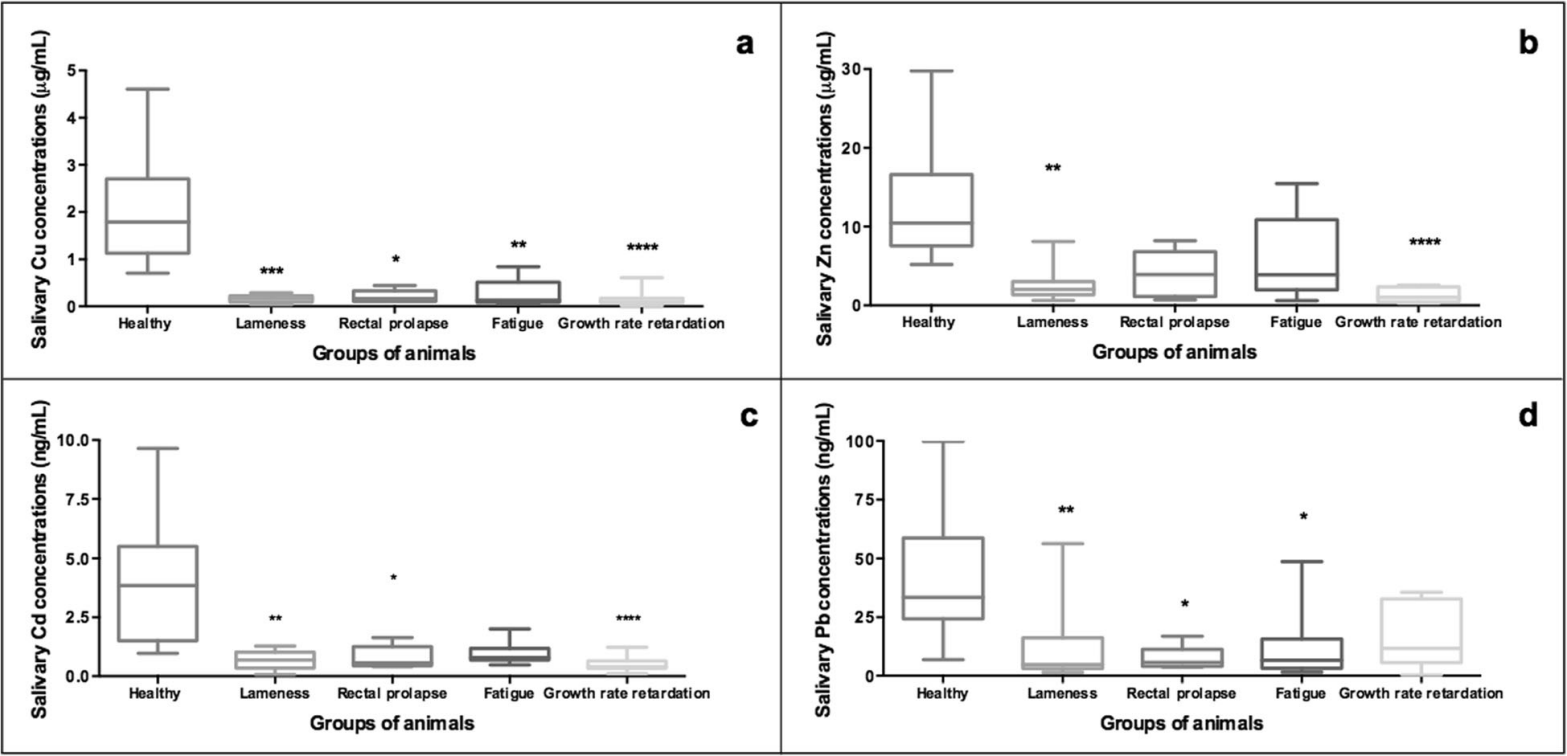
Concentration of the salivary trace elements, Cu (**a**) and Zn (**b**), Cd (**c**) and Pb (**d**), in clinically healthy pigs (*n* = 15) and in pigs suffered from lameness (*n* = 13), rectal prolapse (*n* = 9), fatigue (*n* = 9) and growth rate retardation (*n* = 11). Box-and-whisker plot showing median (horizontal line inside box), mean (plus symbol), 25 and 75 percentiles (edge of box), 10 and 90 percentiles (whiskers) and individual data points beyond (filled circle); significant pairwise comparisons are noted (adjusted p)

The median Cu concentrations were 1.78 μg/mL (25th and 75th percentiles of 1.13 and 2.70, respectively) in clinically healthy pigs, while the median Cu levels in all diseased animals, based on statistical significance, were lower and ranged between 0.101 and 0.163 μg/mL (*p* < 0.05). The group of pigs with growth rate retardation included the group of diseased pigs with lower Cu values (Fig. 2a).

Similar median Zn concentrations were obtained in pigs with fatigue, and the pigs suffered from rectal prolapse with values of approximately 3 μg/mL, which were lower than those observed in clinically healthy pigs (10.45 μg/mL, 25th and 75th percentiles of 7,59 and 16,60, respectively), but without statistical significance. Moreover, clinically healthy animals showed statistically significant higher Zn levels than pigs with lameness and those with growth rate retardation (2.049 μg/mL, 25th and 75th percentiles of 1.36 and 3.04 and 1.029 μg/mL, 25th and 75th percentiles of 0.452 and 2.364, respectively) (Fig. 2b). Similarly, statistically significant lower Cd concentrations were found in pigs suffering from lameness (0.6855 ng/mL), rectal prolapse (0.559 ng/mL) and growth rate retardation (0.408 ng/mL) in comparison to clinically healthy pigs, with median values of 3.85 ng/mL (Fig. 2c). The median Cd concentrations reported for the group of pigs with fatigue were also lower than clinically healthy pigs but without statistical significance.

In the group of pigs without clinical signs of disease, the median Pb concentrations was 33.45 ng/mL (25th and 75th percentiles of 24.30 and 58.75, respectively) with statistically significant low levels of Pb than those detected in animals suffering from lameness (8.030 ng/mL), rectal prolapse (4.68 ng/mL) and fatigue (7.190 ng/mL) (*p* < 0.05) (Fig. 2d). However, the low Pb levels in pigs with growth-rate retardation did not show statistically significant differences compared to the group of clinically healthy pigs.

**Fig. 3:**
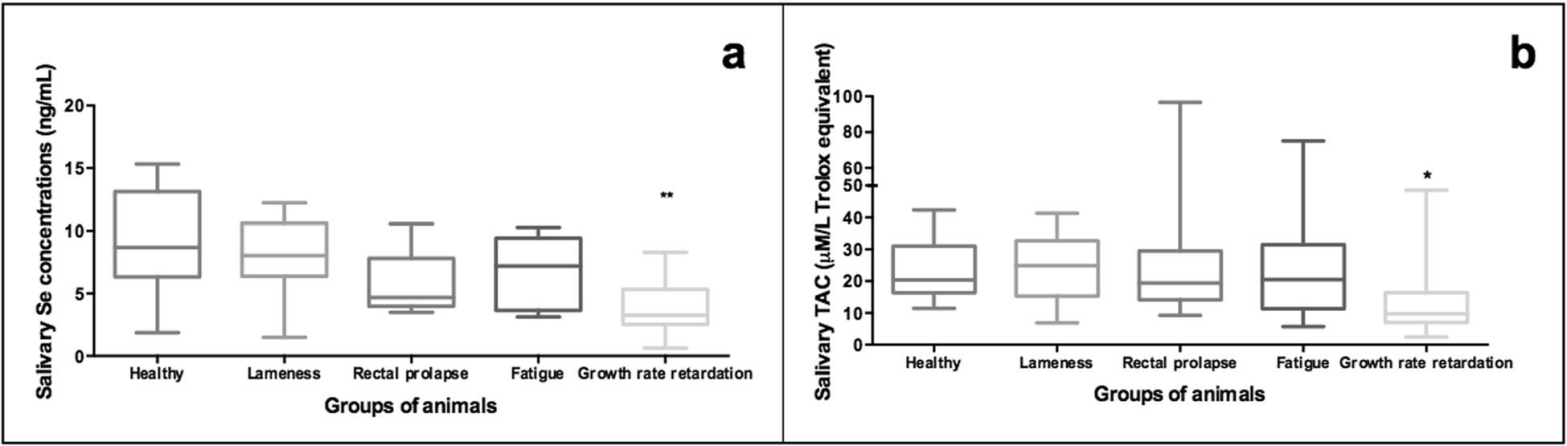
Concentration of the salivary Se (**a**) and TAC (**b**) in clinically healthy pigs (*n* = 15), and in pigs suffered from lameness (*n* = 13), rectal prolapse (*n* = 9), fatigue (*n* = 9) and growth rate retardation (*n* = 11). Box-and-whisker plot showing median (horizontal line inside box), mean (plus symbol), 25 and 75 percentiles (edge of box), 10 and 90 percentiles (whiskers) and individual data points beyond (filled circle); significant pairwise comparisons are noted (adjusted p)

No statistically significant differences were observed in the Se concentrations between the different groups of study with the exception of pigs with growth-rate retardation that showed statistically significant lower values than those observed in clinically healthy pigs (Fig. 3a). Additional file 2 provides raw individual data of trace elements measurements performed in salivary clinical samples of pigs.

### The measurements of acute phase proteins, total antioxidant capacity and adenosine deaminase activity in the clinical samples

TAC measurements showed similar median values in all the studied groups of animals. Despite the lack of statistical significance, lower TAC values were observed in pigs with growth rate retardation (median values of 9.76 mM/L Trolox equivalent, 25th and 75th percentiles of 7.32 and 16.41, respectively) in comparison to clinically healthy pigs (median values of 20.35 mM/L Trolox equivalent, 25th and 75th percentiles of 16.26 and 30.95, respectively) (Fig. 3b).

**Fig. 4:**
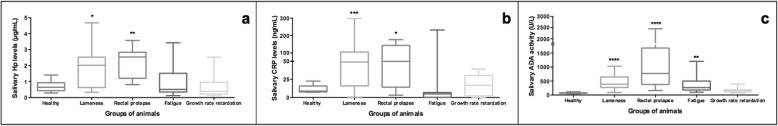
Concentration of the salivary acute phase proteins, Hp (**a**) and CRP (**b**) and ADA (**c**) in clinically healthy pigs (*n* = 15), and in pigs suffered from lameness (*n* = 13), rectal prolapse (*n* = 9), fatigue (*n* = 9) and growth rate retardation (*n* = 11). Box-and-whisker plot showing median (horizontal line inside box), mean (plus symbol), 25 and 75 percentiles (edge of box), 10 and 90 percentiles (whiskers) and individual data points beyond (filled circle); significant pairwise comparisons are noted (adjusted p)

Additionally, the Hp levels detected in the clinically healthy animals (median value 0.65 μg/mL (25th and 75th percentiles of 0.43 and 0.93, respectively)) were similar to those of the animals who suffered from fatigue (0.51 μg/mL) and slightly higher than those observed in animals with growth rate retardation (0.39 μg/mL); otherwise, higher median Hp concentrations were reported in the group of animals with lameness (2.02 μg/mL) and rectal prolapse (2.54 μg/mL) (Fig. 4a).

Similar concentrations were recorded for clinically healthy pigs regarding the CRP values (median value 8.86 ng/mL, 25th and 75th percentiles of 7.63 and 15.77, respectively), alongside animals with signs of fatigue (median value 4.82 ng/mL, 25th and 75th percentiles of 0.15 and 6.84, respectively) and pigs with growth rate retardation (median value 16.80 ng/mL, 25th and 75th percentiles of 2.19 and 30.36, respectively). In contrast, the concentrations of CRP in pigs suffering from lameness (48.88 ng/mL) and rectal prolapse (51 ng/mL) appeared increased compared to clinically healthy pigs (Fig. 4b).

The levels of ADA activity were lower in healthy pigs (median value 65 U/L, 25th and 75th percentiles of 60 and 82, respectively) compared to all groups of diseased animals. However, statistically significant differences were observed between healthy pigs and those with lameness, rectal prolapse and fatigue (Fig. 4c). The highest ADA value was observed in animals suffering from rectal prolapse (median value 773.3 U/L, 25th and 75th percentiles of 380 and 1686, respectively), followed by pigs with lameness (median value 388 U/L, 25th and 75th percentiles of 267 and 646, respectively) and pigs with fatigue (median value 266 U/L, 25th and 75th percentiles of 179 and 499, respectively). Furthermore, the levels of ADA in pigs with growth-rate retardation (median value 156.7 U/L, 25th and 75th percentiles of 110 and 190, respectively) were slightly higher than those reported in healthy pigs without statistical significance. Additional file 3 provides raw individual data of TAC, Hp, CRP and ADA measurements performed in salivary clinical samples of pigs.

**Fig. 5:**
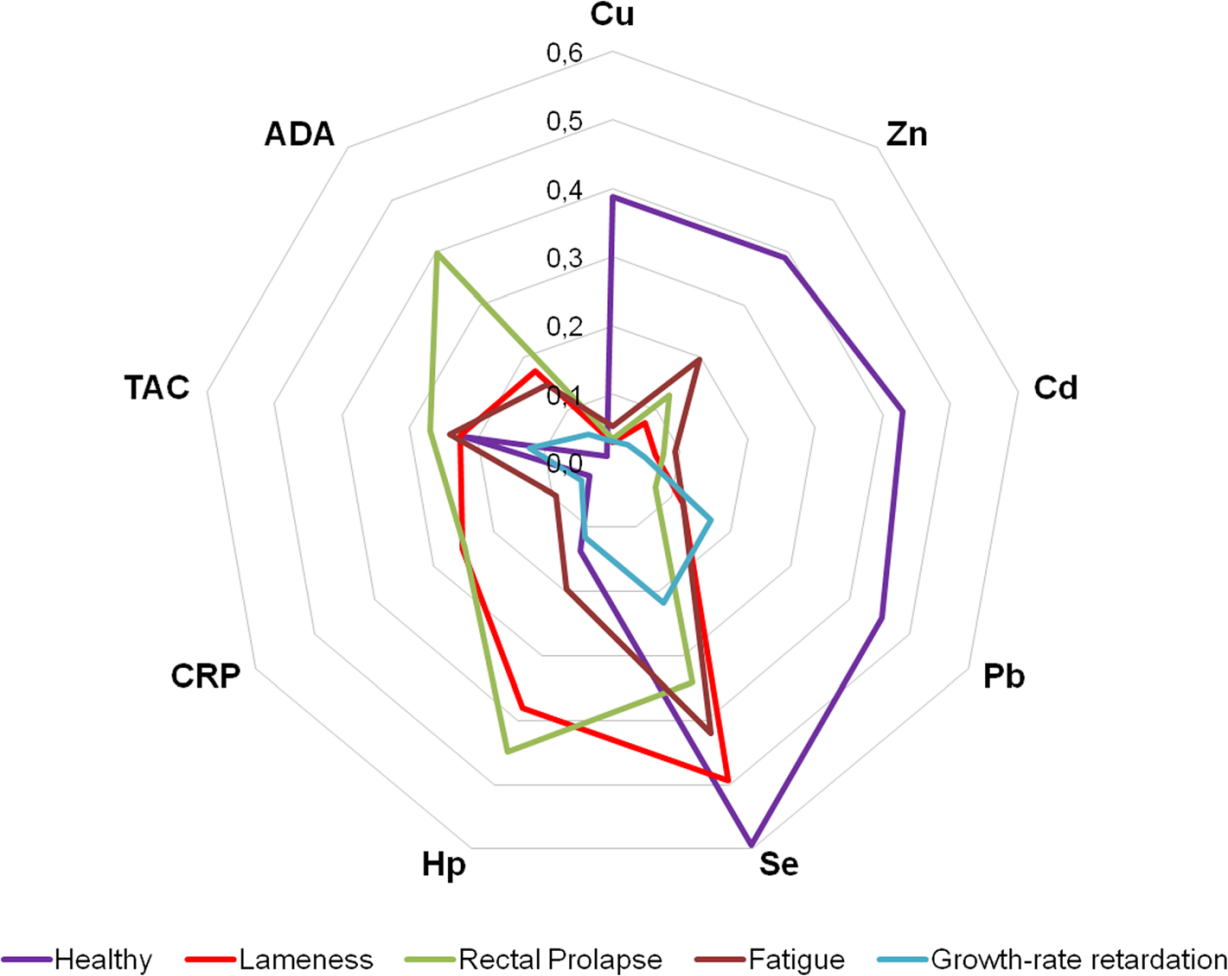
Radar Charts for comparing all the quantitative variables measured (Cu, Zn, Cd, Pb, Se, CRP, Hp, TAC and ADA) in the different groups of pigs. Values normalized according to the followed formula: value in the chart = (value-min)/(max-min)

Overall, the distribution of the different trace elements and health status markers quantified in healthy pigs and pigs with pathologic conditions (lameness, rectal prolapse, fatigue or growth-rate retardation) could be seen in Fig. 5. Moreover, the general shape observed in each health condition was different when all the 9 markers were considered. Some markers were more pronounced in a specific condition, such as Cu or Cd in healthy animals or CRP and Hp in rectal prolapse or lameness, while other markers were lacking, such us ADA or CRP in healthy animals or Cu in all pathological conditions.

### A first approach to the differences in salivary trace element levels between farms

**Fig. 6:**
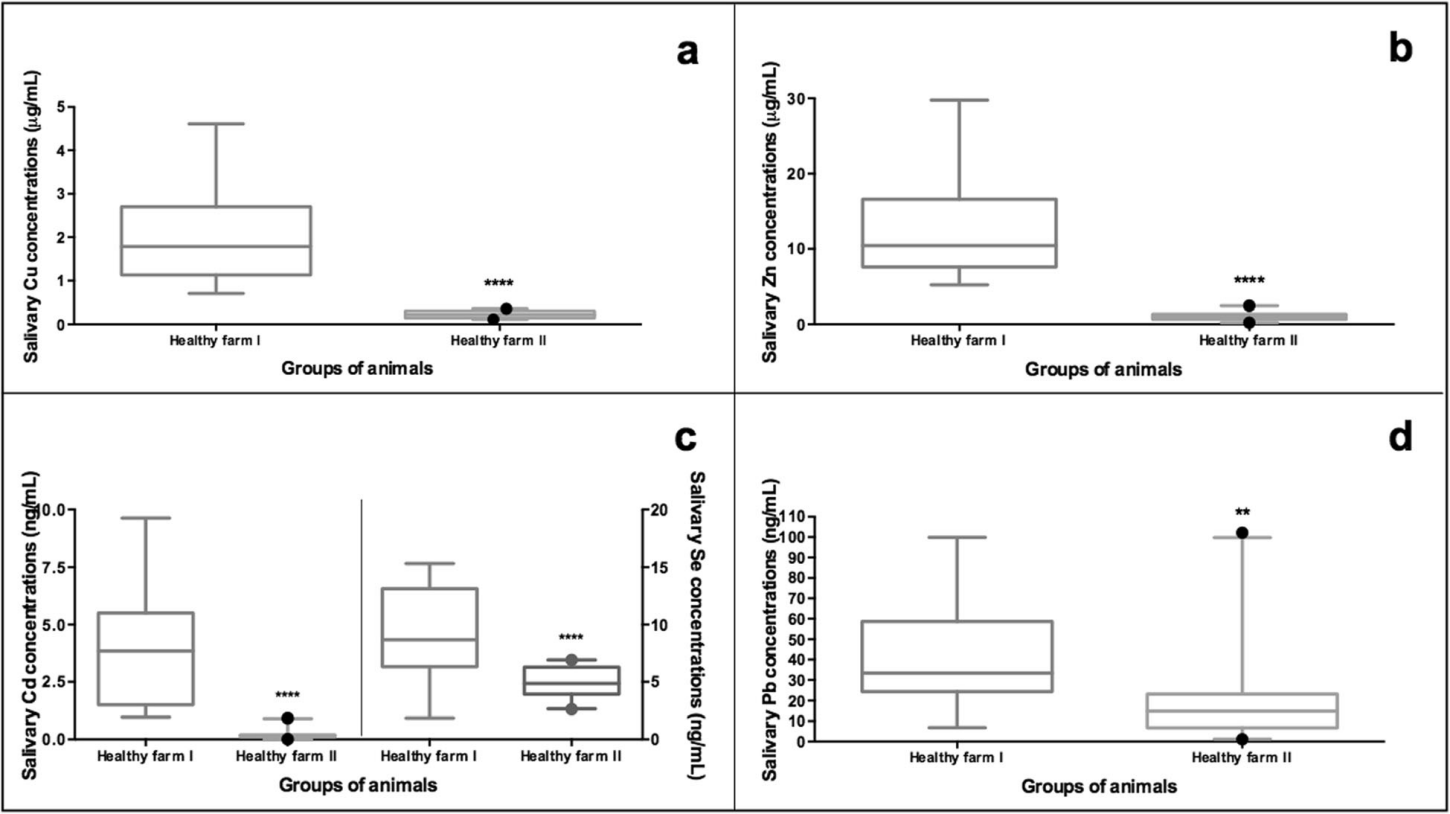
Concentration of the salivary trace elements Cu (**a**), Zn (**b**), Cd and Se (**c**) and Pb (**d**), in clinically healthy pigs from a commercial farm (*n* = 15) and from a farm with documented high sanitary status (*n* = 20). Box-and-whisker plot showing median (horizontal line inside box), mean (plus symbol), 25 and 75 percentiles (edge of box), 10 and 90 percentiles (whiskers) and individual data points beyond (filled circle); significant pairwise comparisons are noted (adjusted p)

The mean concentration of all the studied trace elements was significantly lower in Farm 2, with a documented high sanitary status, compared to the commercial Farm 1 (Fig. 6). However, the levels of the two acute phase proteins studied—Hp (median values of 0.65 vs. 0.55 μg/mL) and CRP (median values of 8.86 vs. 5.02 ng/mL)—and the levels of TAC (median values of 20.35 vs. 17.19 mM/L Trolox equivalent) and ADA (median values of 65 vs. 91 U/L) were similar between the pigs of the two farms. Additional file 4 provides the raw data of measurements performed in saliva samples of healthy pigs from Farm 2.

### Correlation analysis

Statistically significant correlations (*P* < 0.05) were found between the concentrations of the different trace elements analysed and the levels of the health status markers quantified, specifically, Hp with Se and Pb, TAC with Zn, Cu, Cd and Se as well as ADA with Cu, Cd and Pb (Table 3). Moreover, statistically significant positive correlations were also found within all the trace elements quantified except for Se-Pb. No statistical correlations were observed between the acute phase protein CRP and any other trace elements that were studied.

**Table 3:** Correlation coefficients between the trace elements measured and the acute phase proteins and antioxidant status in saliva of pigs. ^*^correlation with statistical significance (*p* < 0.05)

	CRP	Zn	Cu	Cd	Se	Pb	TAC	ADA
Hp	0.59^*^	0.25	0.16	0.10	0.40^*^	−0.29^*^	0.52^*^	0.53^*^
	CRP	−0.11	−0.01	−0.03	0.05	0.01	0.25	0.29^*^
		Zn	0.86^*^	0.77^*^	0.73^*^	0.36^*^	0.67^*^	−0.22
			Cu	0.79^*^	0.60^*^	0.49^*^	0.52^*^	−0.35^*^
				Cd	0.45^*^	0.59^*^	0.38^*^	−0.37^*^
					Se	0.06	0.73^*^	0.09
						Pb	0.08	−0.65^*^
							TAC	0.32^*^

## Discussion

This is the first-time that the trace elements Zn, Cu, Cd, Pb and Se were quantified in the saliva samples of pigs, thus resulting in an analytical validation of the methods used for its quantification in saliva samples being performed. Based on the results, atomic absorption spectrometry has been proved to be sensitive enough for the measurements of Zn, Cu, Cd, Pb and Se in porcine saliva samples. Moreover, good precisions, acceptable accuracies and limits of detection were observed for all the trace elements studied. This report showed the trace element concentrations in porcine saliva for the first time, to the author’s knowledge, thereby warranting additional studies.

Once the validation studies were performed, the possible usefulness of the measurement of trace elements in the saliva samples was analysed for health status assessment in the pigs. Additionally, animals of different health statuses were selected from the same porcine farm to allow for a direct comparison of the trace elements. Furthermore, acute phase proteins, total antioxidant capacity and adenosine deaminase activity were quantified to perform a first health status evaluation of the pigs involved in the study.

The concentrations of Hp and CRP detected in clinically healthy animals were in agreement with previous studies. The median values observed in the present control animals regarding Hp determinations were the same as those reported for male finishing [24] and fattening pigs [25]. Similarly, CRP median values observed in clinically healthy animals were consistent with previous range values reported for male pigs [26]. From the four groups of diseased animals—pigs suffering from lameness, rectal prolapse, fatigue or growth rate retardation—only those with lameness and rectal prolapse showed alteration in the levels of the acute phase proteins compared to clinically healthy animals. The Hp levels detected in pigs with lameness and rectal prolapse were higher than the cut-off value (2.1 μg/mL) reported in animals with PRRS in field conditions [25]. Conversely, the CRP levels observed in these groups of diseased animals were higher than those detected in healthy animals in previous studies as well as the current one [27]; however, they were not as high as the CRP cut-off value (88.52 ng/mL) established for the detection of animals with PRRS [25]. This difference in the magnitude of alteration in the Hp and CRP were in harmony with previous studies performed in serum samples of pigs, in which several sensitivity differences for detecting disease were reported for the different APPs [28], and the incorporation of an APP index was highly suggested [29]. In fact, animals with signs of fatigue and growth rate retardation showed no differences in the median levels of the acute phase proteins in the present study; further studies are necessary to clarify the underlying mechanism of each pathological condition in the field. Thus, only pigs with lameness and rectal prolapse could be classified as suffering from active disease according to our data on acute phase proteins.

TAC levels were quantified in order to obtain further information about the possible oxidative stress implication in each studied disease. Furthermore, the clinically healthy animals showed median salivary TAC values similar to the baseline values recently reported for finishing male pigs [26]. Additionally, the group of pigs suffering from growth-rate retardation showed lower TAC values than those detected in the control pigs, which could indicate that free radicals and the resulting oxidative damage may play an important role in the pathogenesis of the specific process as reported in human medicine [30]; suggestions could include a recommendation for using antioxidant therapy in the treatment of pigs with growth rate retardation, as suggested for lambs [31]. In fact, the involvement of oxidative stress has been evidenced in several porcine pathological conditions such as pneumonia, enteritis or sepsis [32]. However, it has been reported that oxidative stress should be inferred by measuring both oxidants and antioxidants [33]. Therefore, further studies, including a larger number of animals and the quantification of the oxidative damage of each pathological condition, are necessary to corroborate the hypothesis.

Regarding the quantification of the ADA activity, the levels obtained in healthy animals were slightly lower than those previously reported in growing pigs of the same crossbreed origin [5]. Moreover, similar behaviours in diseased pigs than those reported in the mentioned study were observed in the present work where animals with growth-rate retardation showed no statistical differences compared to healthy pigs, while other common pathologies such as lameness, rectal prolapse or respiratory conditions produced an increase—of different magnitudes—in the activity levels of ADA.

When trace elements were quantified, alteration in the median levels in at least one element was observed in all the groups of diseased animals studied when compared to the clinically healthy group of pigs. Furthermore, animals with an evident pathological condition (lameness, rectal prolapse or fatigue) showed low levels of Cu, Zn, Cd or Pb. Moreover, pigs with growth-rate retardation showed exclusively low Se values. These results agree with the low levels of total serum Zn levels previously reported in sepsis [14] and *Actinobacillus pleuropneumoniae* infection [16] with increases after antibiotic treatment [34]. The immunomodulatory function of Zinc makes this metal essential for the immune system [35]; hence, low Zn levels are connected to a higher susceptibility to infections [36]. The active acute phase response observed in our pigs with evident signs of disease—which concurrently showed low salivary Zn values—could be translated to poor immune system activation, specifically in animals with lameness, rectal prolapse and fatigue.

During infection, trace elements are crucial factors since the host limits the number of metals such as Zn, as explained above, while filling up the side of infection with other metals such as copper to obtain an antimicrobial effect [37]. The decreased plasma Zn concentrations have been observed after 4 days of *Actinobacillus pleuropneumoniae* infection in piglets [16] and 1 h of sepsis induction in pigs [37]. Similarly, the total serum Cu levels decreased after sepsis induction with retardation of 3 h compared to Zn [37]. We detected low salivary Zn levels and a significant Cu reduction in all the diseased pigs. Although our study is limited due to the characteristics intrinsic to field conditions and we could not accurately establish the stage of the disease of our animals—according to the acute phase proteins monitored—it could be postulated that pigs with lameness and rectal prolapse were suffering from a persistent pathological condition with a low immune activation, as previously stated. On the other hand, pigs with fatigue and growth-rate retardation did not suffer from active pathological conditions but presented low activation of the immune system. In any case, further studies are guaranteed to elucidate the levels of salivary trace elements throughout the progression of disease in the pig.

Selenium showed exclusively low salivary levels in pigs suffering from growth-rate retardation in line with the low TAC values. These results are in concordance with the involvement of Se as an integral component of glutathione peroxidase, an antioxidant enzyme [38]. Moreover, selenium is known to participate in the antioxidant defence mechanism for the inhibition of viral replication in pigs [20]. Hence, the Se decrease in parallel to the antioxidant status would be expected.

Furthermore, hazardous pollutants, such as Cd and Pb, can affect the immune system of farm animals, and preventive measures should be used to avoid their penetration into the stable environment [39]. Moreover, since the baseline salivary values and the salivary toxic levels for these elements have not been established in pigs until now, the mechanisms to explain the higher Cd and Pb values observed in clinically heathy animals in our study should be explored in depth.

In addition, differences in the concentration of trace elements between clinically healthy animals from different farms were observed in the present study. However, no variations in the concentrations of APP, TAC or ADA were observed between those animals. It has been recently reported that the concentrations of APP and ADA in porcine saliva samples are influenced by the age, sex and management conditions of pigs [26]. Since those characteristics were similar in the two groups of healthy animals included in our study, no differences were expected in APP, TAC and ADA. Therefore, the variations observed in the concentration of trace elements should not be explained by these factors. Consequently, further studies are necessary to understand which factors could influence the levels of trace elements in saliva samples.

Moreover, it is of relevance that although we did not measure the air pollution in each farm—the first farm is situated in the countryside where the pollution levels would be expected to be lower, and the second farm is situated near the dual carriageway where the higher values would be expected—our results show the opposite effect with higher values in the farm situated in the countryside. Therefore, other sources of pollution, such as water or agricultural residues, should be considered in further studies. In addition, the possible influence of the diet composition on the trace elements levels measured in porcine saliva samples has not been stated before and should be studied in the near future.

The combination of all the biomarkers quantified in the present study, performed by radar chart analysis, showed different pathological patterns for each disease condition. A further number of animals within each condition should be analysed to establish the contribution of each marker in disease differentiation. Subsequently, an optimal algorithm, including the best differential markers, could be prepared for its possible implementation for disease monitoring and/or health status assessment in the field.

## Conclusions

Our results identified the salivary levels of the trace elements Zn, Cu, Cd, Pb and Se as promising tools to improve the disease characterisation of pigs in field conditions. However, further large-scale clinical studies are warranted to explain the possible role of the different elements on each pathological condition.

## Methods

### Animals and sampling procedures

Conventional male pigs (Duroc x Landrace x Large White) from a farm in the southeast of Spain were selected for this study. The porcine vaccination program of the farm was: a double vaccination against enzootic pneumoniae, at 7 and 28 days of age, a single vaccination against porcine circovirus, at 28 days of age and a double vaccination against Aujezsky disease, at 11 and 14 weeks of age. All the pigs were housed in pens in groups of a maximum of 10 animals with a total unobstructed floor area available in line with the official standards (Directive 2008/120/EC). Moreover, food and water were available ad libitum. The temperature of the barn was automatically controlled.

The farm was three-phase with several outbreaks of porcine reproductive and respiratory syndrome documented in the last 2 months prior to the beginning of the study. A group of growing pigs (*n* = 42) that showed any clinical sign of disease during the routine veterinary clinical examination was selected for the study. From the total 42 diseased pigs sampled, 21 were of around 119 days of age and the other 21 pigs were younger, of around 75 days. The diseased pigs were divided into different groups according to the signs of disease observed during a routine veterinary clinical examination at the farm: one group included those animals with evident clinical signs of lameness (*n* = 13); a second group was composed of pigs suffering from rectal prolapse (*n* = 9); a third group contained pigs with fatigue symptoms (n = 9); the last group consisted of pigs with retardation in the growth rate without any other signs of disease (*n* = 11). At the time of sampling, pigs without clinical signs of disease were characterised as animals with possible subclinical infections, thus not being selected as control pigs. Therefore, several months after the documented outbreak, clinically healthy animals from the beginning of the growing stage of the production system of around 70 days of age were randomly selected from the same farm and used as control pigs (*n* = 15). Meanwhile, the other group of clinically healthy animals (*n* = 20) from a different farm—in the same commercial company—were also randomly selected for comparison from a pen of growing pigs of similar ages (120 days). This was a closed-cycle farm, with a documented high level of health and sanitary status, and the selected animals were conventional male pigs (Duroc x Landrace x Large White) housed in groups of maximum 10 animals but in closed herds of 10 groups with manual temperature control. The vaccination program and other management conditions were the same as the other farm. Composition of the diets offered to all pigs included in the study (pigs at fattening and finishing stages of the production system) were almost the same (Table 4).

**Table 4:** Composition of the diet offered to commercial Large White x Duroc pigs at the fattening and finishing stage of the production system. ^a^Fattening stage: include pigs between 60 and 100 days of age. Finishing stage: include pigs with more than 100 days of age. ^b^From a premix provided per tn of feed: Cu (from CuSO4·5H2O); Zn (from ZnO2); Se (from Na2SeO3)

Stage^a^	Nutrient and analytical composition (%)								Trace elements composition (g)^b^		
	Crude protein	Fat	Crude fibre	Ash	Starch	Ca	P	Na	Cu	Zn	Se
Fattening	16.5	5.4	3.8	3.9	45.4	0.62	0.44	0.18	15	100	0.34
Finishing	15.5	5.4	3.8	3.9	44.5	0.6	0.43	0.18	15	100	0.30

For the saliva sample collection, the pigs were allowed to chew individual sponges, attached to a thin flexible metal rod, until the sponges were considerably wet; subsequently, the sponges were placed in specific tubes (Salivette tubes, Sarstedt, Nümbrecht, Germany) and centrifuged at 3000 g for 10 min, following which, the saliva samples were aliquoted and stored at − 80 °C until analysis. In each farm, all saliva samples were obtained between 10 a.m. and 11:30 a.m.

All procedures involving animals were approved by the Murcia University Bioethics Committee for Animal Research and followed the recommendations of the European Convention for the Protection of Vertebrate Animals used for Experimental and Other Scientific Purposes (Council of Europe, ETS no. 123). Moreover, the methods used in the experimental phase were aligned with the ARRIVE guidelines and regulations including the oral informed consent of the farm owners prior to their implementation. All animals used in the study continued their routines in the respective growing stages of the production system after the experimental phase. The official veterinary who performed the clinical examination was in charge of the treatment of the diseased animals when necessary.

### Measurements of the trace elements

For trace element quantifications, saliva samples were subjected to acid digestion. Furthermore, for the digestion process, 1 mL of every saliva sample was applied to a specific digest tube, adding 1 mL of nitric acid. After incubation at room temperature for 24 h inside a fume hood, the samples were heated at 120 °C for an hour; after the addition of 1 mL of H_2_O_2_, incubation of 1 h was performed at 120 °C. Finally, the digested samples were cooled at room temperature and filled until 5 mL with pure water. After the digestion, the samples were stored at 4 °C until analyses.

The Zn and Cu levels were measured by flame AAS using a hollow cathode lamp for Cu and Zn, with the wavelength 324.7 nm and 213.8 nm using an air/acetylene flame. The results were expressed as μg/mL.

The levels of Cd, Pb and Se were determined through a graphite furnace AAS using the Zeeman background correction system, graphite tubes with an integrated platform and a hollow cathode lamp for Cd and ultrAA hollow cathode lamps for Pb and Se. Moreover, palladium nitrate was used as matrix modifier for Se measurements. The results were expressed as ng/mL.

Certified Cu, Zn, Cd, Pb and Se standard solutions (Agilent Technologies Spain, Madrid, Spain) were used for constructing the calibration curves of the respective analysis.

### Assessment of intra and inter-assay precision

The intra-assay precision for each trace element was obtained by measuring the concentration of trace element under validation in 2 saliva pools (a pool with high analyte content and the other with low analyte content) 6 times in the same analytical run. Each saliva pool was prepared by mixing an equal amount of saliva from 6 saliva samples with similar trace element concentrations. The inter-assay precision was obtained by measuring a certified standard for each trace element in 5 different days.

Intra- and inter-assay precisions were expressed as the coefficient of variation (CV)—according to the followed formula: CV (%) = (SD/X) * 100—were SD, representing the standard deviation and X the mean value of the different replicates.

### Assessment of assay accuracy

The assay accuracy was calculated indirectly by linearity under dilution. The protocol involved two saliva samples with high levels of each analyte diluted by 0, 6.25, 12.5, 25 and 50%; their concentrations were measured in duplicate. Furthermore, the accuracy of the assay corresponded to the coefficient of correlation between the expected and the observed measurements.

### Assessment of the limit of detection

The limit of detection was assessed by analysing a zero calibrator (assay buffer) 10 times and calculated as the mean concentration obtained, plus 2 wo standard deviations.

### The measurement of acute phase proteins, antioxidant capacity and adenosine deaminase in saliva

The content of Hp and CRP in saliva samples was quantified using time-resolved fluorometric assays, which were previously developed in house and validated [27, 40].

The total TAC was measured in the saliva samples by the ferric reducing antioxidant power assay, which has been previously validated for the porcine saliva sample [26]. The median coefficients of variation for intra and inter assay were below 11% with a high accuracy and a limit of detection of 0.80 μM/L Trolox equivalents.

The levels of ADA activity were quantified in the saliva samples using a commercial human assay (BioSystems S.A., Barcelona, Spain) that has been optimised and validated for use in porcine saliva samples [5].

### Statistical analysis

A normality test, Shapiro-Wilk, was applied to determine whether the results followed a normal distribution pattern. According to the test, the samples had followed a non-normal distribution; therefore, non-parametric Kruskal-Wallis’s multiple comparisons test were applied to search for statistically significant differences between the different groups of animals for all the trace elements, acute phase proteins and antioxidant capacity. The possible difference in marker concentrations between clinically healthy animals from the two different farms was studied using the Man-Whitney statistical test. The Spearman correlation was used for the detection of any statistically significant correlation between the studied markers. Moreover, normality and statistical tests were performed using statistical software (GraphPad Prism 6; GraphPad software Inc., Suite, La Jolla, USA).

The intra- and inter-assay CVs and detection limits were calculated using routine descriptive statistical procedures with Microsoft Excel 2000. Further, an ordinary regression analysis was used to investigate linearity under dilution.

## Supplementary information

**Additional file 1:** Raw data of the analytical validation study performed for trace elements measurements in saliva of pigs.

**Additional file 2:** Raw data of the trace elements measured in saliva samples of pigs under clinical condition.

**Additional file 3:** Raw data of the Hp, CRP, TAC and ADA concentrations measured in saliva samples of pigs under clinical conditions.

**Additional file 4:** Raw data of all the measurements performed in saliva samples of healthy pigs from farm 2.

## Data Availability

All data generated or analysed during this study are included in this published article and its supplementary information files:

## Abbreviations

AAS: Atomic absorption spectrometry
ADA: Adenosine deaminase
APPs: Acute phase proteins
Cd: Cadmium
CRP: C-reactive protein
Cu: Copper
CV: Coefficient of variation
Hp: Haptoglobin
Pb: Lead
PCV2: Porcine circovirus type 2
PRRS: Porcine reproductive and respiratory virus
SAA: Serum amyloid A
Se: Selenium
TAC: Total antioxidant capacity
Zn: Zinc
